# FBXW7 inactivation induces cellular senescence via accumulation of p53

**DOI:** 10.1038/s41419-022-05229-2

**Published:** 2022-09-14

**Authors:** Longyuan Gong, Danrui Cui, Dian Liu, Xiao Shen, Hui Pan, Xiufang Xiong, Yongchao Zhao

**Affiliations:** 1 Department of Hepatobiliary and Pancreatic Surgery, The First Affiliated Hospital, Zhejiang University School of Medicine, Hangzhou, China; 2 Zhejiang Provincial Key Laboratory of Pancreatic Disease, The First Affiliated Hospital, Zhejiang University School of Medicine, Hangzhou, China; 3 Institute of Translational Medicine, Zhejiang University School of Medicine, Hangzhou, China; 4 Cancer Center, Zhejiang University, Hangzhou, China; 5 Department of Lung Transplantation, The First Affiliated Hospital, Zhejiang University School of Medicine, Hangzhou, China; 6 Cancer Institute of the Second Affiliated Hospital, Zhejiang University School of Medicine, Hangzhou, China

**Keywords:** Senescence, Ubiquitin ligases

## Abstract

F-box and WD repeat domain containing 7 (FBXW7) acts as a substrate receptor of SKP1-CUL1-F-box (SCF) E3 ubiquitin ligase and plays crucial roles in the regulation of several cellular processes, including cell growth, division, and differentiation, by targeting diverse key regulators for degradation. However, its role in regulating cellular senescence remains elusive. Here, we found that FBXW7 inactivation by siRNA-based knockdown or CRISPR/Cas9-based knockout induced significant cellular senescence in p53 wild-type cells, but not in p53 mutant or null cells, along with activation of both the p53/p21 and p16^INK4a^/Rb pathways. Simultaneous p53 inactivation abrogated senescence and cell growth arrest induced by FBXW7 deficiency as well as the alteration of both the p53/p21 and p16^INK4a^/Rb pathways. Moreover, *Fbxw7* deletion accelerated replicative senescence of primary mouse embryonic fibroblasts in a p53-dependent manner. In addition, FBXW7 deletion induced the senescence-associated secretory phenotype to trigger secondary senescence. Importantly, in a radiation-induced senescence mouse model, simultaneous deletion of *p53* rescued accelerated senescence and aging caused by *Fbxw7* loss. Thus, our study uncovered a novel role for FBXW7 in the regulation of senescence by eliminating p53.

## Introduction

F-box and WD repeat domain containing 7 (FBXW7), also known as CDC4, is a substrate receptor of SKP1-CUL1-F-box (SCF) E3 ubiquitin ligase. FBXW7 directly recognizes and binds to phosphorylated substrates, such as c-MYC, c-JUN, cyclin E, Notch, and MCL1 [1, 2]. FBXW7 plays a crucial role in the regulation of several vital cellular processes, including cell growth, division, and differentiation. Most substrates regulated by FBXW7 are oncoproteins, and a high mutational rate of FBXW7 has been frequently found in many cancer types, including lung [3, 4], colorectal [5], and breast cancers [6]. Importantly, in multiple Fbxw7 mouse models, tissue-specific *Fbxw7* knockout or mutation knock-in promotes tumorigenesis. For example, mice with deleted *Fbxw7* in the T-cell lineage develop spontaneous thymic lymphoma [7, 8]. Therefore, FBXW7 has been designated as a well-established tumor suppressor [1, 8, 9]. However, FBXW7 was reported to confer radiation survival by targeting the tumor suppressor p53 for degradation [10–12] and regulate chronic myelogenous leukemia (CML) via a p53-dependent apoptosis pathway [13], which adds another layer of complexity to the role of FBXW7 in tumorigenesis. Most recently, an outstanding study showed that downregulating FBXW7 promotes chondrocyte senescence and osteoarthritis development [14]. Given that p53 plays a critical role in the induction of senescence by inducing p21 expression [15], an inhibitor of cell-cycle progression, FBXW7 may regulate senescence by controlling p53 protein levels.

Cellular senescence is a progressive and phenotypically diverse state of irreversible cell-cycle arrest that occurs in proliferating cells in response to various stresses, such as replicative exhaustion, oncogene activation, DNA damage, and cell–cell fusion [16]. Accumulating evidence has implicated senescence in an array of physiological and pathological processes, such as embryonic development, wound healing, degenerative disorders, aging, and cancer [17]. Given that senescence is an irreversible growth arrest, it generally inhibits tumor growth. However, accumulating senescent cells in tissues may also contribute to the development of tumors by modulating the tissue microenvironment, suggesting that senescence plays a dual role in the development of tumors [18]. Senescent cells exhibit a set of general hallmarks, including enlarged and flattened morphology, cell-cycle arrest, increased senescence-associated β-galactosidase (SA-β-Gal) levels, and senescence-associated secretory phenotype (SASP) [19]. In response to various stresses and stimuli, both p53 and Rb serve as hubs in the central activation pathways of senescence [20]. Activated p53 triggers the expression of p21, responsible for G1 cell-cycle transition, and further causes cell-cycle arrest, a typical hallmark of senescence. Besides p21, p53 induces E2F7 expression, which represses many E2F target genes, subsequently leading to senescence [15]. Previous studies have shown that FBXW7 interacts with and targets p53 for proteasomal degradation in both stressed and unstressed cells [10–12], suggesting the possibility that FBXW7 modulates senescence in a p53-dependent manner.

In this study, we report that FBXW7 inactivation readily induces cellular senescence in p53 wild-type cells, but not in p53 mutant or null cells, along with activation of both the p53/p21 and p16^INK4a^/Rb pathways. Simultaneous p53 knockdown or knockout not only abrogated cellular senescence induced by FBXW7 inactivation but also rescued the cell growth arrest. Moreover, in a radiation-induced senescence mouse model, simultaneous deletion of *p53* rescued the increased senescence phenotype in *Fbxw7*^*+/−*^ mice. Thus, our study uncovered a novel role for FBXW7 in the regulation of senescence by eliminating p53.

## Results

### FBXW7 silencing induces senescence in cancer cells harboring wild-type p53 along with activation of both p53/p21 and p16^INK4a^/Rb pathways

**Fig. 1:**
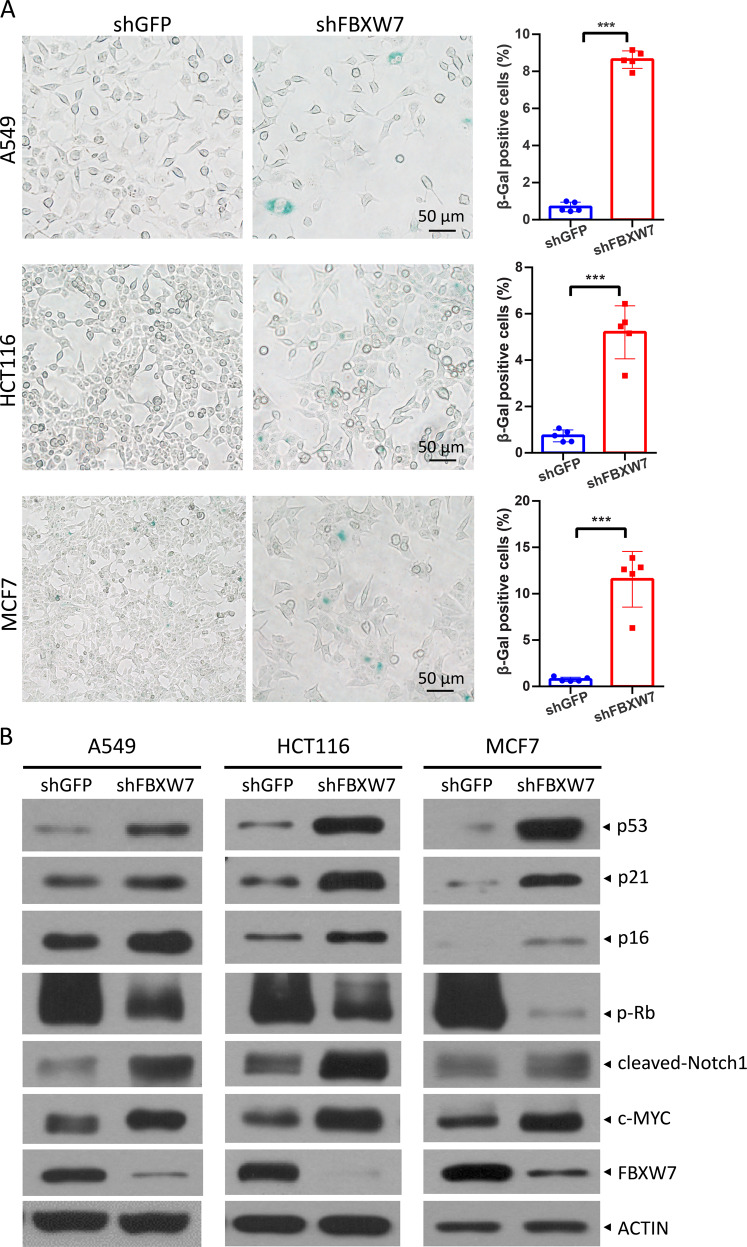
FBXW7 silencing induces senescence in cancer cells harboring wild-type p53 along with activation of both p53/p21 and p16^INK4a^/Rb pathways. A549, HCT116, and MCF7 cells were infected with a lentivirus expressing shFBXW7 or shGFP, selected by puromycin for seven days, and subjected to senescence-associated β-galactosidase (SA-β-Gal) staining, followed by microscopy (**A** left) or immunoblotting (IB) with the indicated antibodies (**B**). The percentage of SA-β-Gal-positive cells in the total number of cells was determined (**A** right). Scale bars represent 50 μm. Data are presented as the mean ± standard deviation (SD). ***p < 0.001.

We previously found that FBXW7 binds to phosphorylated p53, leading to p53 ubiquitination and proteasomal degradation [10]. Given that p53 plays a critical role in the induction of senescence, we investigated whether FBXW7 could regulate senescence by modulating p53 levels in this study. Indeed, multiple human cancer cells harboring wild-type p53, including A549, HCT116, MCF7, SJSA, and H460 cells, displayed an enlarged and flattened cell morphology (Fig. 1A and Fig. S1), a general senescence hallmark, upon FBXW7 knockdown, indicating that senescence was induced. The percentage of SA-β-Gal was dramatically increased in FBXW7 knockdown cells (Fig. 1A and Fig. S1B). Moreover, we determined cell senescence using Sudan-Black-B (SBB) staining, an additional reliable approach to detect senescent cells [21]. Likewise, upon FBXW7 knockdown, the positive SBB staining was obviously increased, as reflected by increased intracellular blue-black granules (Fig. S1C). These data further confirmed that senescence had occurred. Interestingly, FBXW7 knockdown had no effect on cell morphology or the percentage of SA-β-Gal-positive cells in DLD-1 and MDA-MB231 cells with mutant p53 and p53-null H1299 cells, respectively (Fig. S2), suggesting that FBXW7 knockdown induces senescence in a p53-dependent manner. Senescent cell-cycle arrest is regulated by the p53/p21 and p16^INK4a^/Rb tumor suppressor pathways [22]. Next, we measured the levels of these crucial factors when the cell morphology was flattened after FBXW7 knockdown. Indeed, FBXW7 knockdown significantly increased the levels of p53, p21, and p16, as well as cleaved-Notch1 and c-MYC, two well-known substrates of FBXW7, while decreasing the levels of phosphorylated Rb, suggesting that both the p53/p21 and p16^INK4a^/Rb tumor suppressor pathways were activated (Fig. 1B).

### Simultaneous p53 deletion abrogates the senescence induced by FBXW7 deletion

**Fig. 2:**
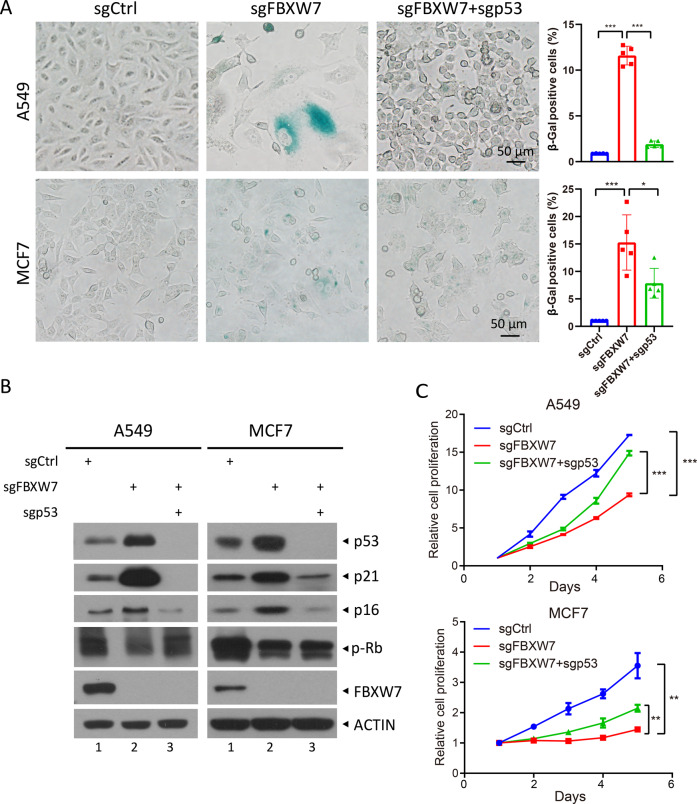
Simultaneous *p53* deletion abrogates cell senescence induced by *FBXW7* deletion. A549 and MCF7 cells with deletion of the indicated genes using CRISPR/Cas9 technology were subjected to SA-β-Gal staining, followed by microscopy (**A** left) or IB with the indicated antibodies (**B**), or cell counting kit (CCK)-8-based cell proliferation assay (**C**). The percentage of SA-β-Gal-positive cells in the total number of cells was determined (**A** right). Scale bars represent 50 μm. Data are presented as the mean ± SD. *p < 0.05, ***p < 0.001. Cell proliferation is expressed as a fold-change compared to day 1. Data are presented as the mean ± standard error of the mean (SEM) from three independent experiments. *n* = 3; **p < 0.01, ***p < 0.001.

To explore the underlying mechanism of FBXW7-induced senescence, we simultaneously silenced p53, c-MYC, and Notch1 in FBXW7 knockdown A549 and MCF7 cells (Fig. S3A), as c-MYC and cleaved-Notch1, which are involved in oncogene-induced senescence [23, 24], were accumulated in FBXW7 knockdown senescent cells (Fig. 1B). We found that simultaneous p53 knockdown rescued the senescence phenotype caused by FBXW7 deficiency (Fig. S3B). Interestingly, although there are multilevel crosstalks between p53 and Notch [25], only p53, but not Notch1, knockdown reversed senescence induced by FBXW7 deficiency, suggesting induced senescence independent of Notch1. Furthermore, we generated FBXW7 and p53 deleted cells via a CRISPR/Cas9-based approach and found that *FBXW7* deletion alone induced obvious senescence, while deletion of both *FBXW7* and *p53* suppressed the induction of senescence (Fig. 2A). Consistently, *FBXW7* deletion caused an increase in the levels of p53, p21, and p16 and a decrease in Rb phosphorylation, which was rescued by simultaneous *p53* deletion (Fig. 2B, lanes 3 versus 2). In addition, simultaneous *p53* deletion partially rescued the cell growth arrest caused by *FBXW7* deletion, which further confirmed that senescence was rescued (Fig. 2C). Collectively, these results indicate that p53 accumulation by FBXW7 inactivation plays a causal role in the promotion of senescence and cell growth arrest.

### Fbxw7 deletion accelerates replicative senescence of primary mouse embryonic fibroblasts (MEFs) by inducing p53 expression

**Fig. 3:**
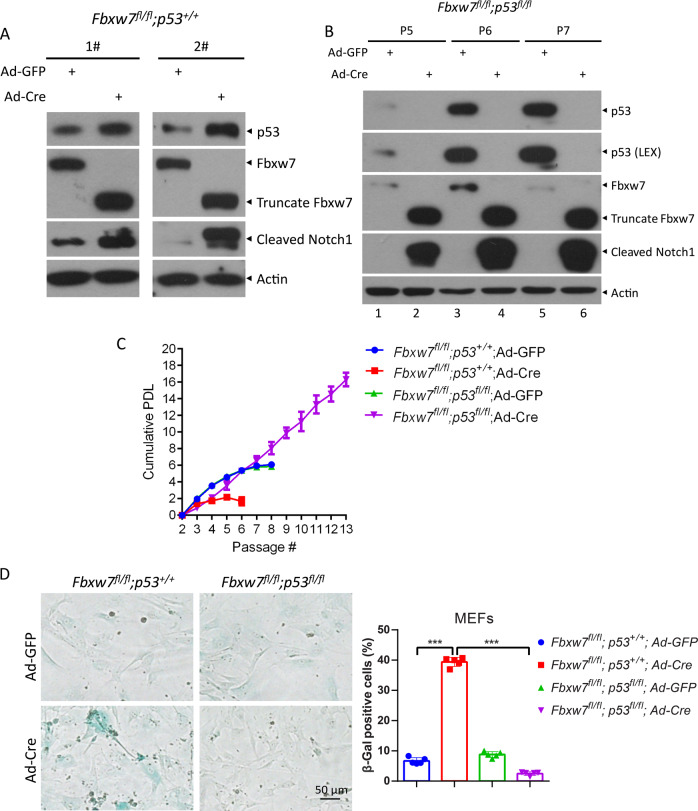
*Fbxw7* deletion accelerates the senescence of primary mouse embryonic fibroblasts (MEFs) by inducing p53 expression. **A** Two independent primary *Fbxw7*^*fl/fl*^*;p53*^*+/+*^ MEFs were infected with the adenovirus-expressing Cre recombinase (Ad-Cre) or adenovirus-expressing green fluorescent protein (Ad-GFP) for 72 h, and then subjected to IB with the indicated antibodies. **B** Primary *Fbxw7*^*fl/fl*^*;p53*^*fl/fl*^ MEFs were infected with Ad-Cre or Ad-GFP, then passaged for indicated times, followed by IB with the indicated antibodies. **C**, **D** Primary *Fbxw7*^*fl/fl*^*;p53*^*+/+*^ and *Fbxw7*^*fl/fl*^*;p53*^*fl/fl*^ MEFs were infected with Ad-Cre or Ad-GFP, followed by cell passage on a 3T9 protocol to measure the cumulative population doubling level (**C**), or SA-β-Gal staining at P6 (**D** left). The percentage of SA-β-Gal-positive cells in the total number of cells was determined (**D** right). Scale bar represents 50 μm. Data are presented as the mean ± SD. ***p < 0.001.

To investigate whether the FBXW7–p53 axis is involved in the senescence of normal cells, we passaged primary MEFs to induce replicative senescence. First, we generated primary *Fbxw7*^*fl/fl*^*;p53*^*+/+*^ and *Fbxw7*^*fl/fl*^*;p53*^*fl/fl*^ MEFs by crossing *Fbxw7*^*fl/+*^*;p53*^*fl/+*^ mice. Consistently, *Fbxw7* deletion caused significant p53 accumulation in two pairs of primary *Fbxw7*^*fl/fl*^*;p53*^*+/+*^ MEFs that had been infected with adenovirus-expressing Cre recombinase (Ad-Cre) to delete *Fbxw7* (Fig. 3A). Furthermore, the protein levels of p53 were gradually increased during passaging in *Fbxw7*^*fl/fl*^*;p53*^*fl/fl*^ MEFs expressing Fbxw7 and p53 as wild-type MEFs after infection with adenovirus-expressing GFP (Ad-GFP) (Fig. 3B, lanes 5 and 3 versus 1), implying a significant role of p53 in replicative senescence. We then measured the cumulative population doubling time of MEFs by serial passaging using a 3T9 protocol and found that *Fbxw7*^*fl/fl*^*;p53*^*+/+*^ MEFs underwent senescence at P4 after *Fbxw7* deletion by Ad-Cre and *Fbxw7*^*fl/fl*^*;p53*^*+/+*^ and *Fbxw7*^*fl/fl*^*;p53*^*fl/fl*^ MEFs underwent senescence at P6 upon Ad-GFP infection (Fig. 3C), whereas *Fbxw7*^*fl/fl*^*;p53*^*fl/fl*^ MEFs were immortalized without senescence upon *Fbxw7* and *p53* deletion by Ad-Cre (Fig. 3B, lanes 2, 4, and 6; Fig. 3C). Meanwhile, the percentage of SA-β-Gal-positive cells was dramatically increased in *Fbxw7*^*fl/fl*^*;p53*^*+/+*^ MEFs upon *Fbxw7* deletion at P6, which was completely reversed by simultaneous deletion of p53 in *Fbxw7*^*fl/fl*^*;p53*^*fl/fl*^ MEFs (Fig. 3D). Taken together, these results demonstrate that *Fbxw7* loss accelerates replicative senescence by inducing p53 expression.

### FBXW7 deletion promotes senescence and aging in a p53-dependent manner in vivo

**Fig. 4:**
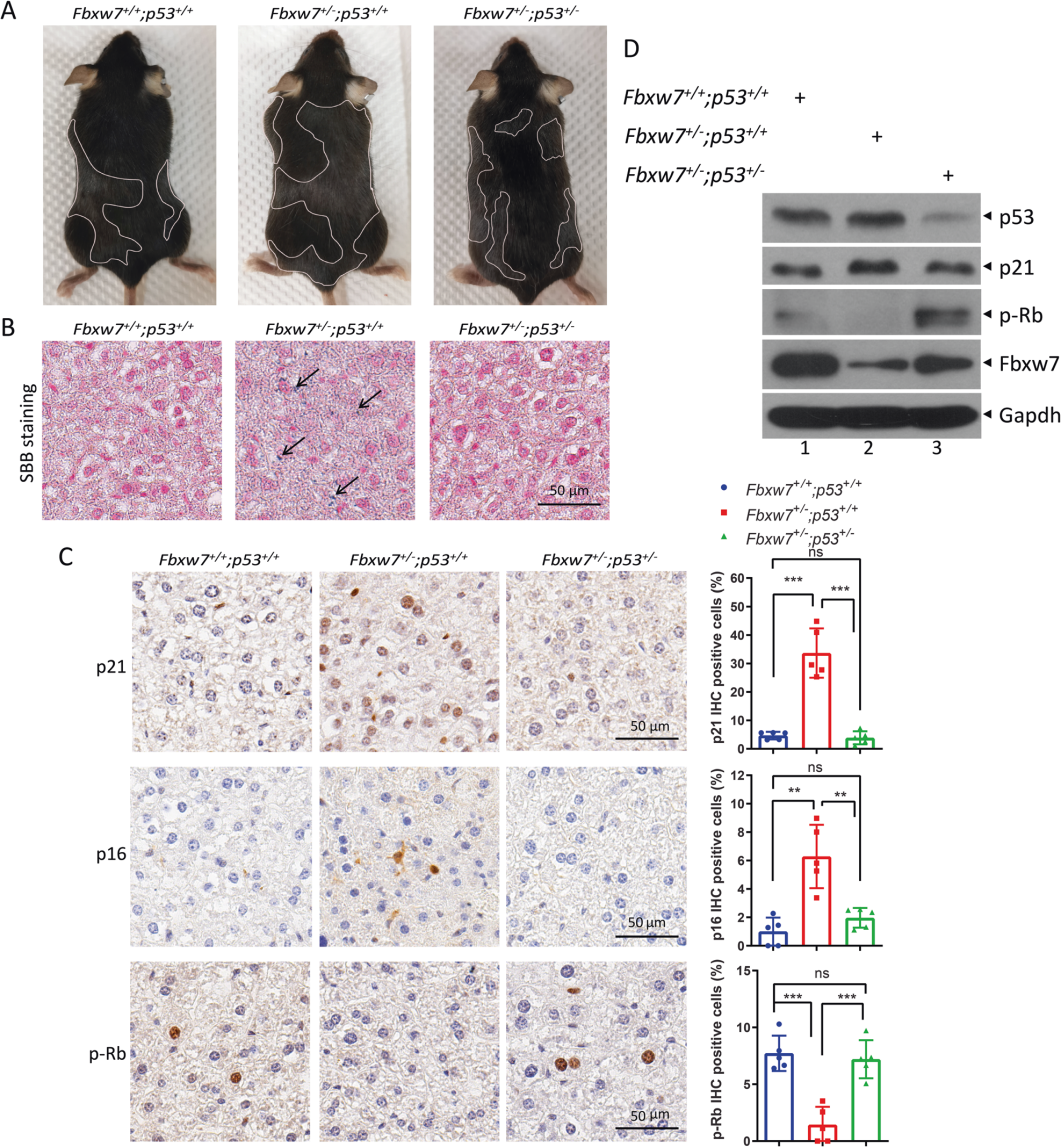
FBXW7 deletion promotes senescence in a p53-dependent manner in vivo. Three littermate mice with the indicated genotypes were exposed to 7 Gy irradiation and maintained for 3 months, followed by being photographed (**A**). Liver tissues of mice were harvested and subjected to SBB staining (**B**), immunohistochemistry (IHC) (**C** left), or IB with the indicated antibodies (**D**). The area of graying hair is circled by a white line. The percentage of staining-positive cells in the total number of cells was determined from at least five random fields of liver tissues (**C** right). **p < 0.01; ***p < 0.001; ns not significant. Scale bars represent 50 μm.

Homozygous loss of *Fbxw7* causes embryonic lethality, while mice with *Fbxw7* heterozygous deletions survive, although Fbxw7 is a haplo-insufficient gene [26–28]. We explored the in vivo role of Fbxw7 in the regulation of senescence using Fbxw7 heterozygous loss mice (*Fbxw7*^*+/−*^) combined with *p53*^*+/−*^ mice. We administered 7 Gy ionizing radiation to trigger premature aging in female mice with three genotypes (*Fbxw7*^*+/+*^*;p53*^*+/+*^, *Fbxw7*^*+/−*^*;p53*^*+/+*^, and *Fbxw7*^*+/−*^*;p53*^*+/−*^) [29]. Initially, the mice exhibited almost no systemic reactions. However, after three months, three littermates with the three genotypes showed representative hair graying, which is a typical sign of aging in animals and humans (Fig. 4A). Given that cellular senescence is usually linked to aging [30], the hair graying of *Fbxw7*^*+/−*^*;p53*^*+/+*^ mice was the most serious, indicating that the most senescence is induced upon *Fbxw7* heterozygous deletion, which can be reversed by simultaneous *p53* heterozygous loss. Moreover, the strongest SBB staining of liver tissues from *Fbxw7*^*+/−*^*;p53*^*+/+*^ mice further confirmed that the most senescence is induced upon *Fbxw7* heterozygous deletion (Fig. 4B). In addition, both immunohistochemistry and /or immunoblotting of liver tissues collected from the littermates showed that compared to *Fbxw7*^*+/+*^*;p53*^*+/+*^ mice, the levels of p21 and p16 were significantly increased, while the phosphorylation of Rb was decreased in *Fbxw7*^*+/−*^*;p53*^*+/+*^ mice, indicating that more cells were undergoing senescence (Fig. 4C, D). Consistently, the protein levels of p53 were also increased in the liver tissues of *Fbxw7* heterozygous mice (Fig. 4D, lanes 2 versus 1). Simultaneous *p53* heterozygous loss also reversed the induction of p21 and p16, and the reduction in Rb phosphorylation (Fig. 4C,D, lanes 3 versus 2). Collectively, these results indicate that *Fbxw7* deletion promotes senescence in a p53-dependent manner in vivo. Based on these results, we conclude that FBXW7 deficiency induces senescence both in vivo and in vitro via p53 induction.

### FBXW7 deletion induces senescence-associated secretory phenotype (SASP)

**Fig. 5:**
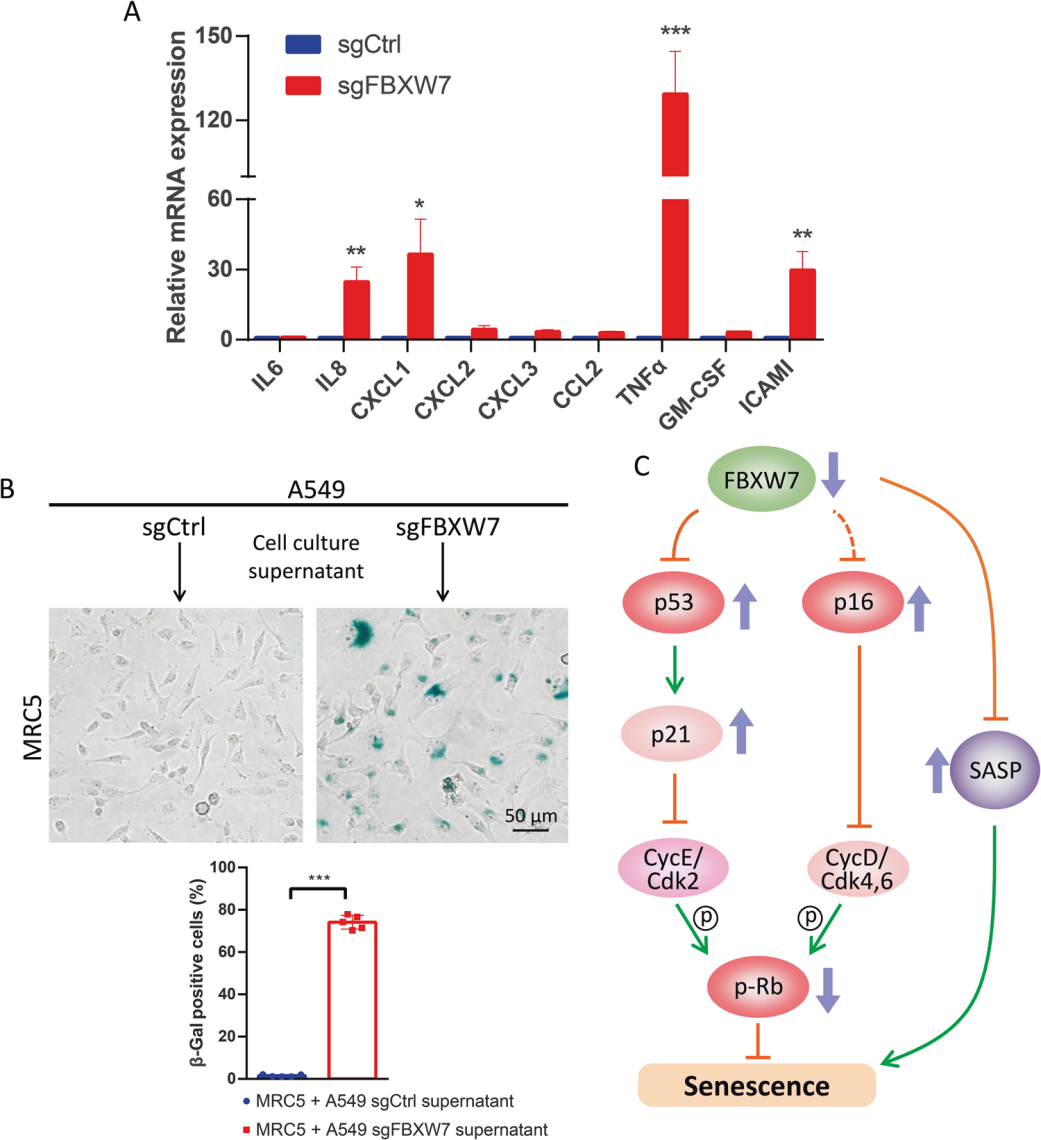
FBXW7 loss induces the senescence-associated secretory phenotype (SASP). **A** A549 cells, with or without FBXW7 deletion, were harvested for qRT-PCR analysis for the indicated SASP factors. Data are presented as the mean ± SEM from three independent experiments. *n* = 3; *p < 0.05; **p < 0.01; ***p < 0.001. **B** The conditional medium from A549 cells, with or without FBXW7 deletion, was used to culture MRC5 cells for 2 weeks, followed by SA-β-Gal staining (top). The percentage of SA-β-Gal-positive cells in the total number of cells was determined (bottom). Scale bar represents 50 μm. Data are presented as the mean ± SD. ***p < 0.001. **C** A model for FBXW7 inactivation-induced senescence.

Senescent cells secrete various factors, including pro-inflammatory cytokines and chemokines, growth modulators, angiogenic factors, and matrix metalloproteinases, to communicate with their microenvironment and influence the surrounding cells, termed SASP or senescence messaging secretome [31, 32]. Next, we investigated whether *FBXW7* deletion led to the development of SASP. First, we measured the levels of a set of factors involved in the senescence process, including interleukin (IL6), chemokines (IL8, CXCL1-3, and CCL2), other inflammatory factors (TNFα and GM-CSF), and the ligand (ICAM1). Among them, IL8, CXCL1, TNFα, and ICAM1 were the most fluctuating factors in *FBXW7-*deleted senescent cells (Fig. 5A). Furthermore, conditioned media from A549 sgCtrl and sgFBXW7 cells were collected to culture human fetal lung fibroblast MRC5 cells for 2 weeks. We found that MRC5 cells cultured with the conditioned medium from A549 sgFBXW7 cells displayed an enlarged and flattened morphology with increased SA-β-Gal-positive staining (Fig. 5B), indicating the occurrence of secondary senescence. These results suggest that FBXW7 deletion-induced senescence may regulate the microenvironment to influence the surrounding cells by developing SASP.

## Discussion

FBXW7 acts as a substrate receptor of SCF ubiquitin ligase and plays critical roles in multiple cellular processes, such as cell proliferation and differentiation, by targeting many key regulators for degradation [2, 9, 33]. In this study, we found that FBXW7 inactivation promoted the senescence of p53 wild-type cancer and normal cells and that p53 induction played a causal role in FBXW7 deficiency-induced cellular senescence both in vitro and in vivo. The major findings of this study can be listed as follows: (1) flattened cell morphology and enhanced staining of SA-β-Gal were observed in various types of cancer and normal cells harboring wild-type p53 upon FBXW7 deficiency; (2) simultaneous p53 inactivation by siRNA-based knockdown or CRISPR/Cas9-based knockout abrogated the senescence induced by FBXW7 deficiency; (3) in an in vivo radiation-induced senescence model, heterozygous loss of *Fbxw7* promoted hair graying and cell senescence in liver tissues, whereas the heterozygous loss of *p53* reversed these alterations; and (4) many SASP factors secreted by FBXW7-deficient A549 cells induced senescence of MRC5 cells.

Given that FBXW7 is a well-established tumor suppressor, FBXW7 inactivation should theoretically promote cancer cell growth. Unexpectedly, in our study, FBXW7 inactivation via siRNA-based knockdown or CRISPR/Cas9-based knockout significantly induced senescence in lung cancer (A549 and H460), colon cancer (HCT116), breast cancer (MCF7), and osteosarcoma (SJSA) cells, suggesting that this is a common phenomenon (Fig. 1 and Fig. S1). Mechanistically, p53, a novel substrate of FBXW7 [10–12], plays a causal role in FBXW7 deficiency-induced cellular senescence both in vitro and in vivo. A recent outstanding study reported that downregulating FBXW7 promotes chondrocyte senescence and osteoarthritis development upon mechanical overloading by targeting MKK7 for degradation, which consequently stimulates the JNK signaling [14]. In addition, FBXW7 was reported to positively regulate the function of XRCC4 and EGFR by promoting XRCC4 polyubiquitination via the K63 linkage [34], and stabilizing EGFR [35], respectively. Inactivation of XRCC4 [20] or EGFR [36] induces cellular senescence. Moreover, Fbxw7 deletion in MEFs upregulates the transcription of p16^INK4a^ and p19^Arf^ to induce cell-cycle arrest [37]. Given simultaneous p53 deletion cannot completely abrogate the senescence and proliferation induced by FBXW7 deletion in cancer cells (Fig. 2), it is worth to further exploring other mechanisms contributing to FBXW7-regulated senescence, such as via MKK7. However, another recent study proposed that FBXW7 promotes senescence and pulmonary fibrosis, a typical age-related disease, by targeting the telomere protein TPP1 for degradation and accelerating the uncapping of telomere [38]. The seemingly contradictory roles of FBXW7 in the regulation of senescence may be attributed to the diverse substrates whose degradation is subject to FBXW7 regulation in a cell- or tissue-context-dependent manner.

As a general tumor suppressor in cancer, FBXW7 is inactivated by mutations, with an overall mutation frequency of approximately 6% [39]. Upon FBXW7 inactivation, accumulated oncogenic substrates, such as c-MYC, cyclin E, and Notch, may play major roles in tumorigenesis triggered by FBXW7 inactivation. Nevertheless, cellular senescence promoted by FBXW7 inactivation may also contribute to tumorigenesis in a particular process by reprograming the microenvironment by promoting the secretion of SASP factors, which warrants further investigation. Notch1, rather than SASP alone, is required for secondary senescence [40]. Although we found that the simultaneous silencing of Notch1 could not rescue FBXW7 inactivation-induced senescence (Fig. S3B), given that Notch1 is a well-known substrate of FBXW7, it is necessary to investigate whether FBXW7 inactivation contributes to secondary senescence by inducing Notch1 expression.

Primary MEFs are canonical models to study senescence via replicative arrest through the accumulation of the INK4 family like p16^INK4a^ [41]. p53 inactivation alone is insufficient to immortalize primary MEFs [42]. Interestingly, in our study, *Fbxw7* deletion alone induced premature senescence in primary MEFs, whereas both *Fbxw7* and *p53* deletion immortalized primary MEFs (Fig. 3C, D). We speculate that p53 deletion coordinates with the accumulation of oncogenic substrates of FBXW7, such as c-MYC, which release primary MEFs beyond the Hayflick Limit [43], leading to immortalization.

Senescent cells accumulate in multiple tissues with age, which is one of the causative processes of aging and aging-associated diseases. In addition to aging, subsequent studies reinforced the importance of cellular senescence as a safeguard against cancer [44]. The physiological relevance of cellular senescence is not limited to tumor suppression, but also includes many biological processes, such as embryonic development [45, 46], wound healing [47, 48], tissue repair [49], organismal aging [50], and various endocrine diseases [51]. Cellular senescence can favor the damage repair of the body, but can also cause harm to the body via the microenvironment [52]. In addition, accumulating senescent cells in tissues may promote the development of tumors by modulating the microenvironment via SASP [18]. Thus, cellular senescence acts as a double-edged sword, with both beneficial and detrimental effects on physiological and pathological processes. Future investigations of the precise role of senescence may be beneficial for senolytic therapy.

In summary, our study uncovered the significant role of FBXW7 in the regulation of cellular senescence and revealed the two underlying mechanisms associated with it. First, upon FBXW7 inactivation, p53 is directly accumulated, and then, increased p53 transactivates p21, subsequently inhibiting the cyclin E/Cdk2 activity. Second, FBXW7 inactivation increases p16 expression via an unknown mechanism, which further represses the activities of cyclin D/Cdk-4,6. As a result, Rb phosphorylation is significantly inhibited and dephosphorylated Rb represses the transcription of genes required for cell-cycle progression by directly binding to the transactivation domain of E2F, finally leading to the induction of senescence. Moreover, FBXW7 inactivation-induced senescent cells secrete many SASP factors that promote secondary senescence (Fig. 5C).

## Methods

### Cell culture

Human cancer cells, including A549, HCT116, MCF7, SJSA, H460, H1299, DLD-1, and MDA-MB231, obtained from American Type Culture Collection (ATCC), were cultured at 37 °C in a humidified incubator with 5% CO_2_. A549, MCF7, SJSA, H1299, DLD-1, and MDA-MB231 cells were maintained in Dulbecco’s modified Eagle’s medium (DMEM) supplemented with 10% (v/v) fetal bovine serum (FBS) and 1% penicillin/streptomycin (P/S). H460 cells were maintained in RPMI-1640 medium supplemented with 10% FBS and 1% P/S. HCT116 cells were maintained in McCoy’s 5 A medium supplemented with 10% FBS and 1% P/S.

### Lentiviral production and generation of stable cell lines

Short hairpins targeting FBXW7 (targeting sequence: 5′-ACA GGA CAG TGT TTA CAA A-3′), p53 (targeting sequence: 5′-CAC CAT CCA CTA CAA CTA CAT-3′), c-MYC (targeting sequence: 5′-CAG TTG AAA CAC AAA CTT GAA-3′), and Notch1 (targeting sequence: 5′-CCG GGA CAT CAC GGA TCA TAT-3′) were subcloned into pLKO.1-puro vector. Lentivirus expressing shRNA was produced by transfecting shRNA vector, psPAX2, and pMD2.G plasmids into 293 T cells. Cells were infected by lentivirus and then selected for stable expression with puromycin for seven days.

### CRISPR/Cas9-based FBXW7 and p53 knockout

CRISPR/Cas9-based FBXW7 or/and p53 knockout cells were generated as described previously [53]. Briefly, single-guide RNA (sgRNA) was subcloned into the plasmid, pSpCas9(BB)-2A-Puro (PX459). Cells were simultaneously transfected with sequence-verified CRISPR plasmids expressing sgRNA targeting FBXW7 or p53, and selected with puromycin for three days. Then cells were seeded in 100 mm dish at 0.5 × 10^3^, 1 × 10^3^, 2 × 10^3^ cells and cultured for 10–15 days. About 100 single clones were picked under a microscope and were further expanded. The FBXW7, p53, or FBXW7 and p53 double knockout clones were confirmed by immunoblotting and genomic DNA sequencing. The sequences of the sgRNA were used as follows: sgFBXW7: 5′-AAA GTT GGA CCA TGG TTC TG-3′; sgp53: 5′-GCA GTC ACA GCA CAT GAC GG-3′.

### Generation and maintenance of MEFs

Primary MEFs were isolated from day E13.5 embryos and cultured in DMEM supplemented with 15% FBS, 0.1 mM MEM nonessential amino acids, and 1% P/S, and incubated at 37 °C in a 5% CO_2_ humidified incubator.

### MEF cell passage on a 3T9 protocol

Primary MEFs (P1) were infected with adenovirus (Ad-Cre) for 72 h. A total of 9 × 10^5^ cells were seeded in a 60-mm dish, cultured for 72 h, and then counted at each passage until there are not enough cells to subculture. PDL (population doubling level) was calculated using a standard formula: cumulative PDL = initial PDL + 3.32 × [log (current cell yield) − log (cell plated)].

### Immunoblotting and antibodies

Cells were lysed in lysis buffer [50 mM Tris–Cl (pH 7.5), 150 mM NaCl, 1 mM DTT, 1 mM EDTA, 0.5% sodium deoxycholate, 50 mM NaF, 1 mM Na_3_VO_4_, 1% NP-40, 0.1% SDS] with protease inhibitors and phosphatase inhibitors, followed by SDS-PAGE gel electrophoresis and antibody incubation [54]. The target proteins were visualized by chemiluminescence. Antibodies used are listed as follows: FBXW7 (A301-720A, Bethyl Laboratories), p53 (OP43 from Calbiochem for human sample, 2524 and 32532 from Cell Signaling Technology for mouse sample), p21 (2947 from Cell Signaling Technology for human sample, ab188224 from abcam for mouse sample), p16 (92803 from Cell Signaling Technology for human sample, ab211542 from abcam for mouse sample), p-Rb (8516, Cell Signaling Technology), cleaved-Notch1 (4147, Cell Signaling Technology), c-MYC (5605, Cell Signaling Technology), ACTIN (A5441, Sigma), and GAPDH (2118, Cell Signaling Technology).

### SA-β-galactosidase staining for senescence

The staining of senescence-associated β-galactosidase was conducted according to the manufacturer’s instructions (C0602, Beyotime). Briefly, cultured cells were fixed by β-galactosidase staining fixative at room temperature for 15 min, and then incubated in the staining working solution for ~12–24 h at 37 °C. The stained cells were observed and photographed under a light microscope.

### Sudan-Black-B (SBB) staining

Cells adhered to the glass slides were immersed in Sudan-black stain for 2 min, washed with 75% ethanol to remove excess stain, and then immersed in distilled water for 1 min, and counterstained with nuclear fast red stain for 1 min. For paraffin sections of tissues, after deparaffinization and rehydration, the sections were stained according to the procedure for cell staining. The slides were sealed with glycerin gelatin and then observed and photographed in time.

### CCK8 assay

Cells were seeded in 96-well plates in triplicate at 3000 cells per well. Cell proliferation was evaluated by a CCK8 assay according to the manufacturer’s instructions (HY-K0301, MedChemExpress), and results were expressed as the fold-change compared with the control.

### Mouse studies

*Fbxw7*^*+/−*^ mice were generated by crossing *Fbxw7*^*fl/fl*^ (Jackson laboratory, Stock No: 017563) mice with Vasa-Cre (Jackson laboratory, Stock No: 018980) transgenic mice. Mice were genotyped using the following primers to detect floxed Fbxw7 (497 bp), deleted Fbxw7 (662 bp), and WT (315 bp): Fbxw7-cKO-F: 5′-ATT GAT ACA AAC TGG AGA CGA GG-3′; Fbxw7-cKO-R: 5′-ATA GTA ATC CTC CTG CCT TG GC-3′; Fbxw7-KO-F: 5′-GGC TTA GCA TAT CAG CTA TGG-3′. *p53*^*+/−*^ mice were obtained and genotyped as previously described [55]. For the animal studies, all procedures were approved by Zhejiang University Laboratory Animal Center. Animal care was provided in accordance with the principles and procedures outlined in Chinese National Research Guide for the Care and Use of Laboratory Animals.

### Mice radiation exposure

Female mice aged 6–8 weeks were exposed to 7 Gy of radiation (X-RAD 160; PXi) and maintained for 3 months, followed by being photographed and sacrificed for liver tissue collection.

### Immunohistochemical staining

For immunohistochemistry (IHC), 5 μm thick sections of mouse liver tissues were stained with the antibodies, as previously described [56]. The following antibodies were used: p21 (ab188224, abcam), p16 (ab211542, abcam), and p-RB (Ser807/811) (8516, Cell Signaling Technology).

### Quantitative RT-PCR

Quantitative RT-PCR analysis was performed as described previously [57]. Briefly, total RNA was isolated from cells using TRIzol reagent (15596018, Invitrogen). cDNA was generated from RNA using the Prime-Script RT reagent kit (RR037A, Takara), following the manufacturer’s instructions. Quantitative real-time PCR (qRT-PCR) was performed using SYBR Premix Ex Taq (RR420A, TaKaRa) on an Applied Biosystems StepOnePlus^TM^ Real-Time PCR instrument. Relative expression levels of indicated genes were determined using the comparative Ct (2^ΔΔCt^) method with GAPDH as an endogenous normalization control. The following primers were used for qRT-PCR analysis: IL6 forward: 5′-ACT CAC CTC TTC AGA ACG AAT TG-3′, reverse: 5′-CCA TCT TTG GAA GGT TCA GGT TG-3′; IL8 forward: 5′-TTT TGC CAA GGA GTG CTA AAG A-3′, reverse: 5′-AAC CCT CTG CAC CCA GTT TTC-3′; CXCL1 forward: 5′-TCA TTG TGA AGG CAG GGG AA-3′, reverse: 5′- AAT TAA GCC CCT TTG TTC TAA GCC -3′; CXCL2 forward: 5′-AGC TCT CCT CCT CGC ACA-3′, reverse: 5′- GAG TGT GGC TAT GAC TTC GGT-3′; CXCL3 forward: 5′-CGC CCA AAC CGA AGT CAT AG-3′, reverse: 5′-GCT CCC CTT GTT CAG TAT CTT TT-3′; CCL2 forward: 5′- CAG CCA GAT GCA ATC AAT GCC-3′, reverse: 5′-TGG AAT CCT GAA CCC ACT TCT-3′; TNFα forward: 5′-CCT CTC TCT AAT CAG CCC TCT G-3′, reverse: 5′-GAG GAC CTG GGA GTA GAT GAG-3′; GM-CSF forward: 5′- GCT GCT GAG ATG AAT GAA ACA GTA-3′, reverse: 5′-AGC AGT CAA AGG GGA TGA CAA-3′; ICAMI forward: 5′-ATG CCC AGA CAT CTG TGT CC-3′, reverse: 5′-GGG GTC TCT ATG CCC AAC AA-3′; GAPDH forward: 5′-AGG GCA TCC TGG GCT ACA C-3′, reverse: 5’-GCC AAA TTC GTT GTC ATA CCA G-3′.

### Statistical analysis

Statistical analyses were performed by two-tailed Student’s *t*-test, using Statistical Program for Social Sciences software 20.0. Data are expressed as mean ± standard error of the mean (S.E.M) or standard deviation (SD). The statistical significance was set as *p* < 0.05.

## Supplementary information

Supplementary Figure 1

Supplementary Figure 2

Supplementary Figure 3

Supplementary Figure legends

Original IBs

aj-checklist

## Data Availability

The authors declare that all data supporting the findings of this study are available with the article or from the corresponding author upon reasonable request.
