# Supplementary figures and images for: FBXW7 inactivation induces cellular senescence via accumulation of p53
Source: Cell Death Dis. 2022 Sep 14;13(9):788. doi: 10.1038/s41419-022-05229-2 (PMC9475035; doi:10.1038/s41419-022-05229-2)

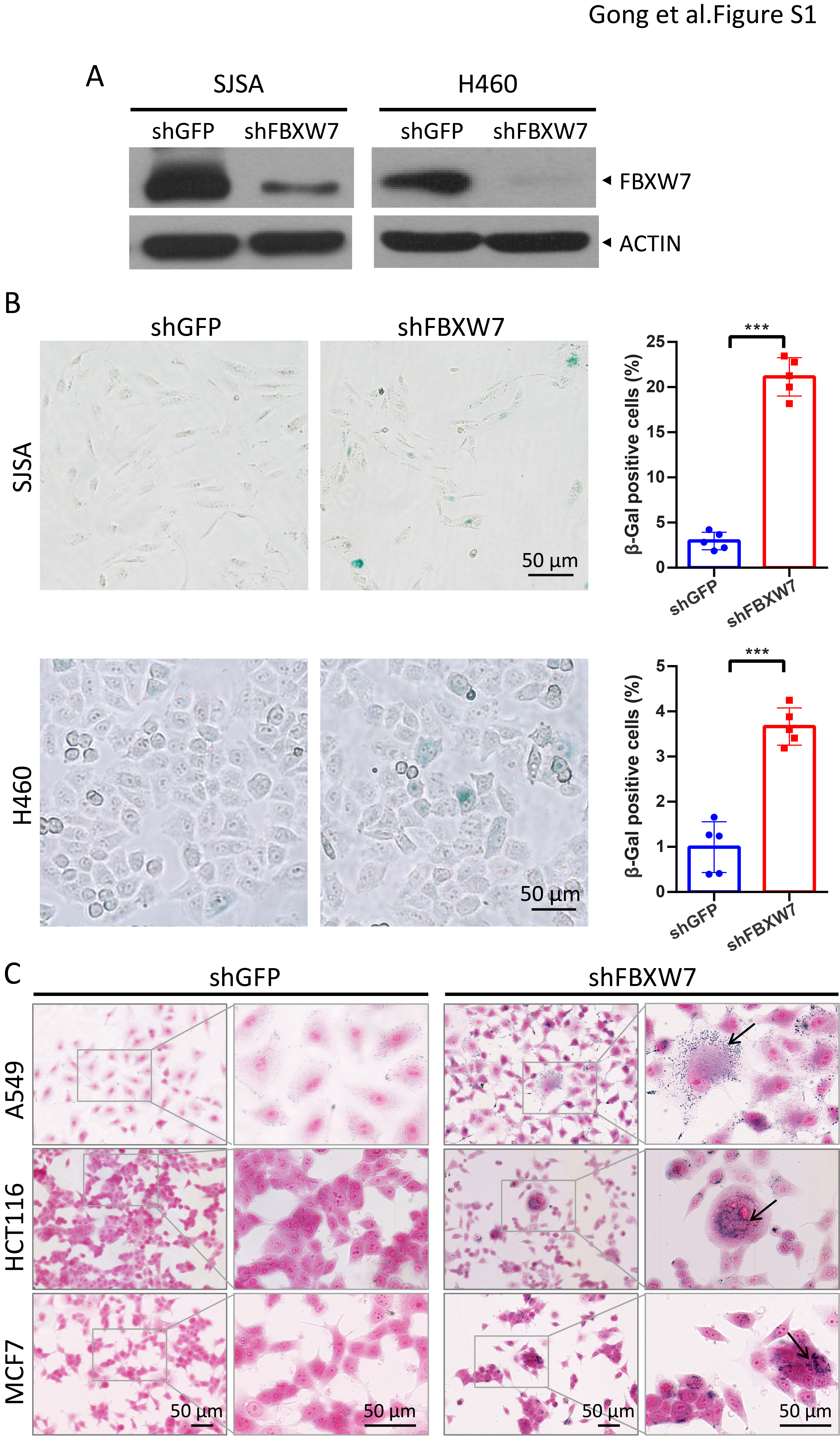

Supplement: Supplementary file 1 — Supplementary Figure 1 [file 41419_2022_5229_MOESM1_ESM.png]

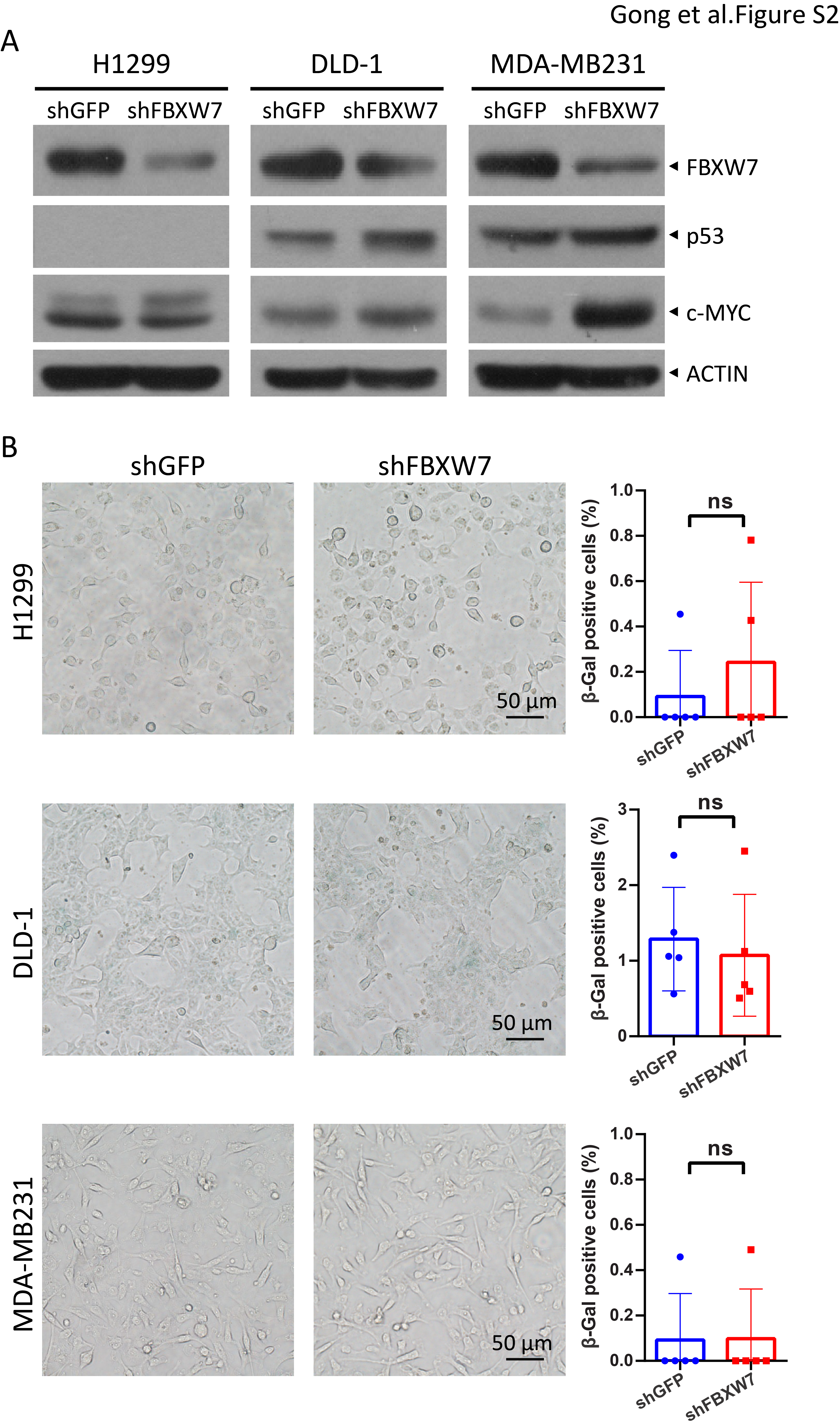

Supplement: Supplementary file 2 — Supplementary Figure 2 [file 41419_2022_5229_MOESM2_ESM.png]

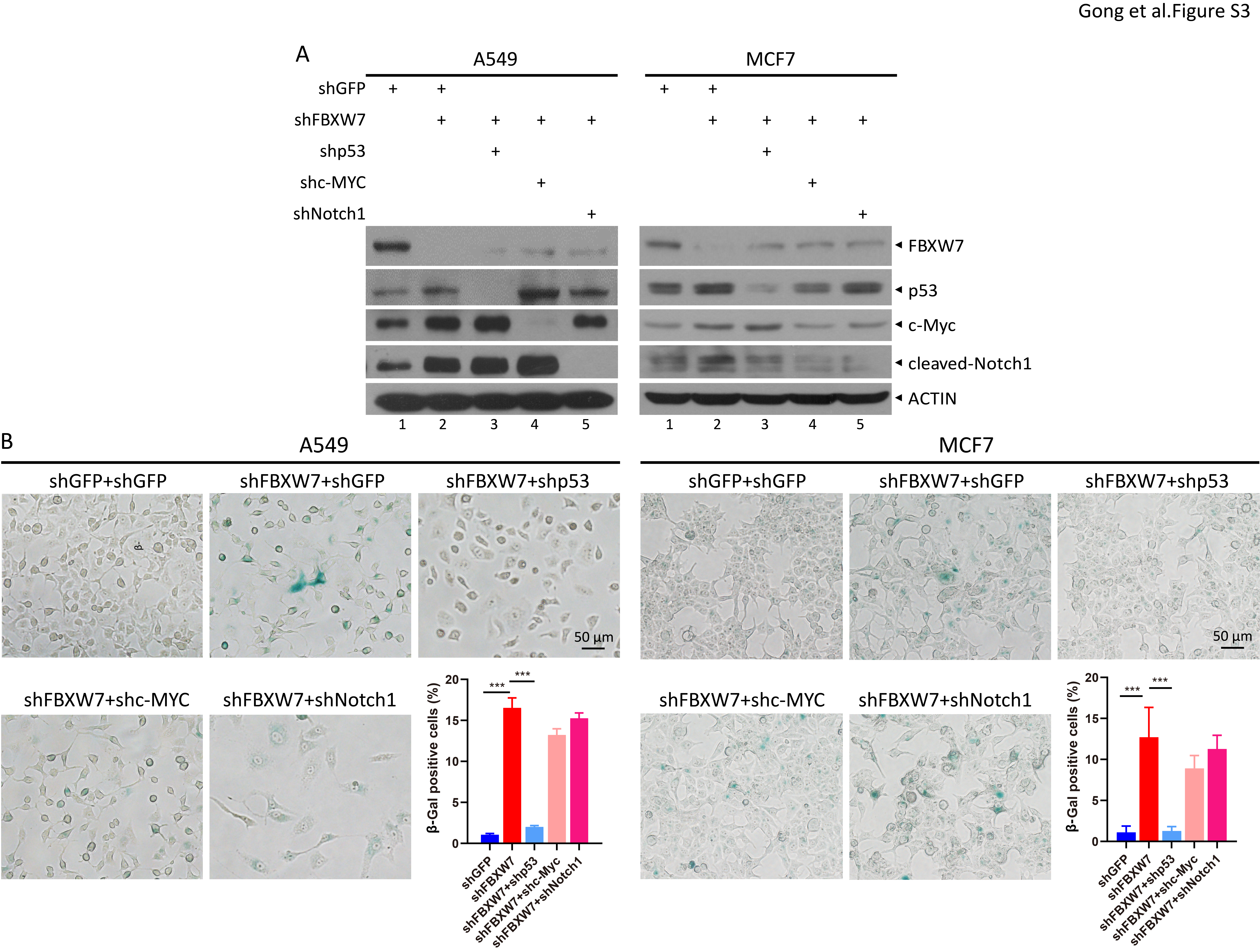

Supplement: Supplementary file 3 — Supplementary Figure 3 [file 41419_2022_5229_MOESM3_ESM.png]

### Figure 1B

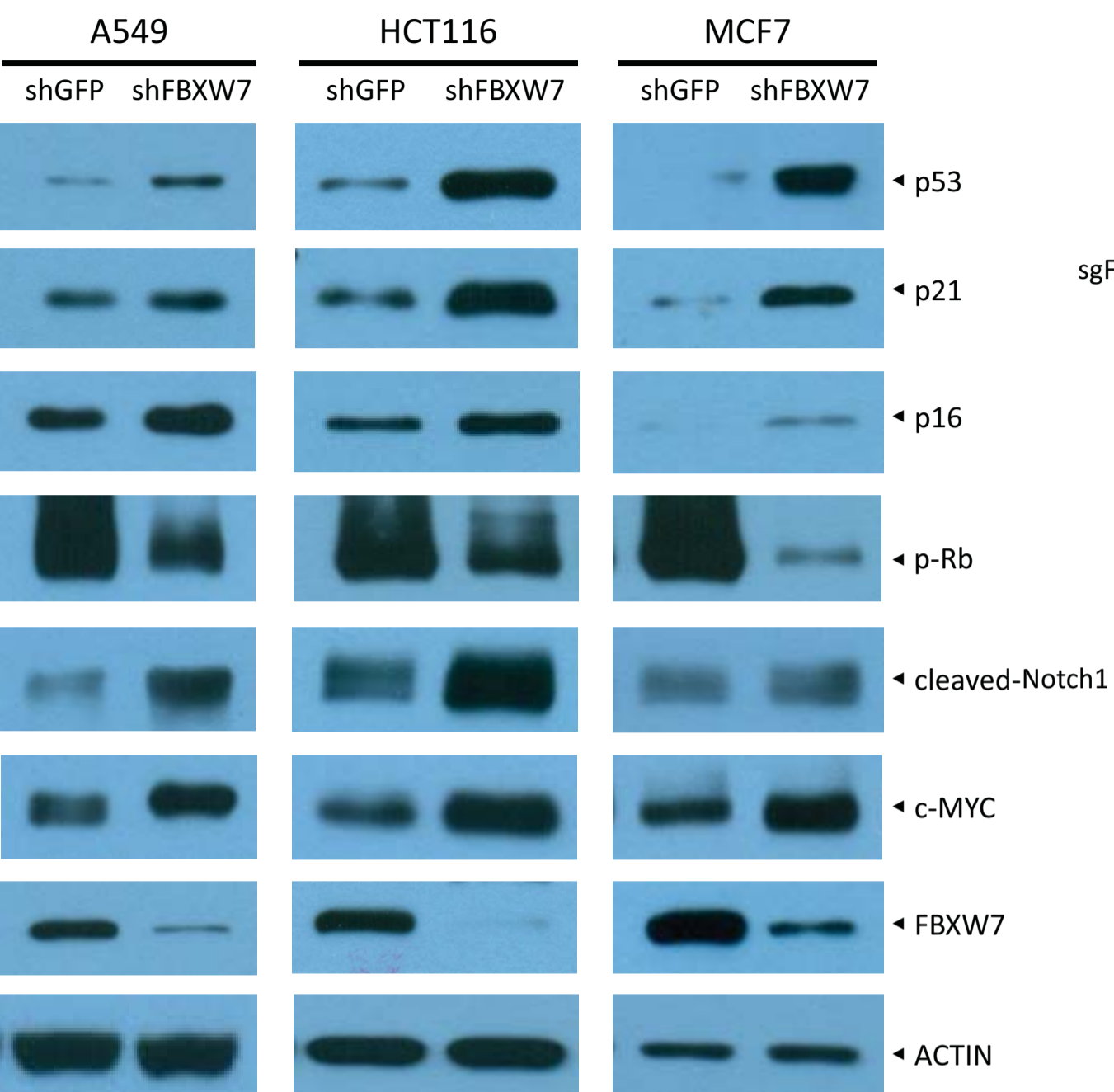

### Figure 2B

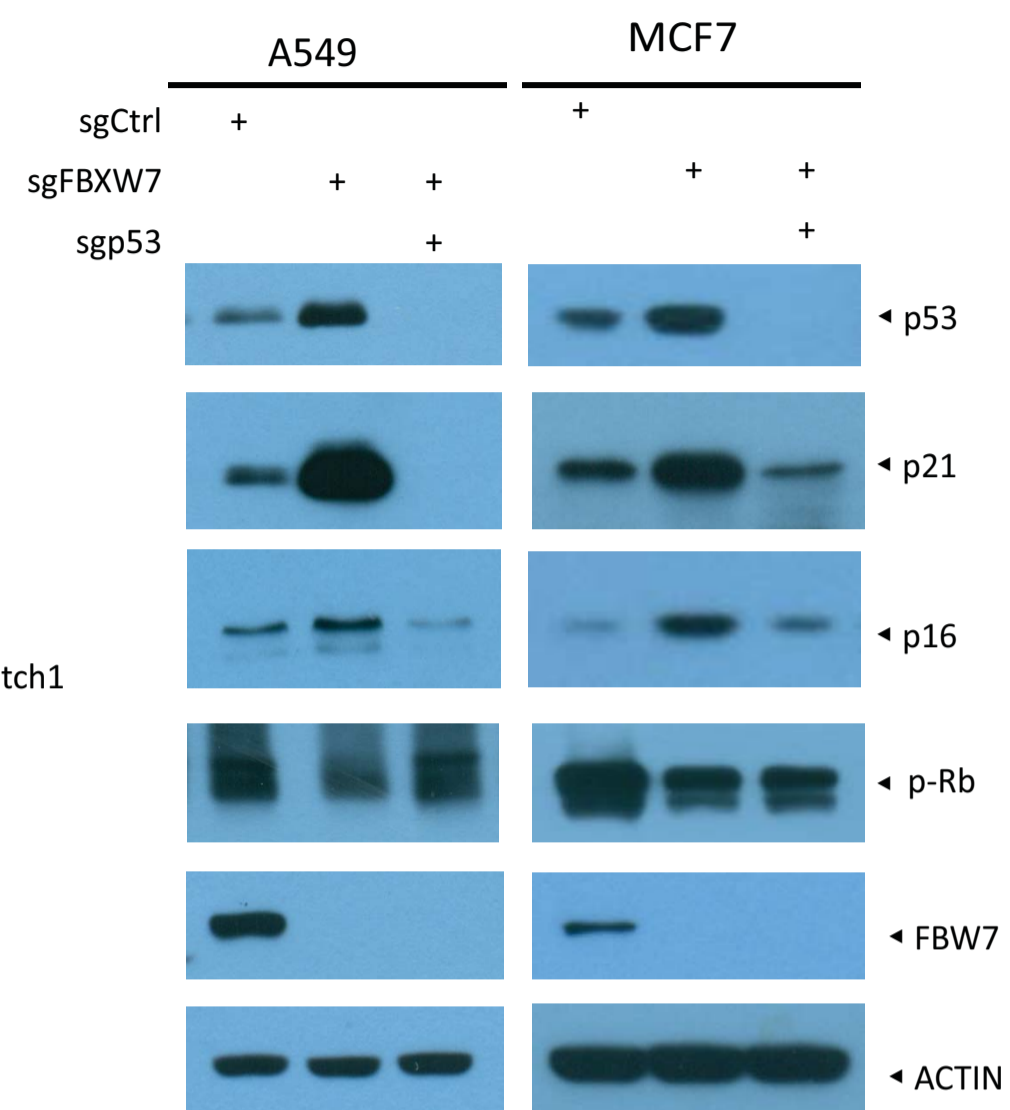

### Figure 3A

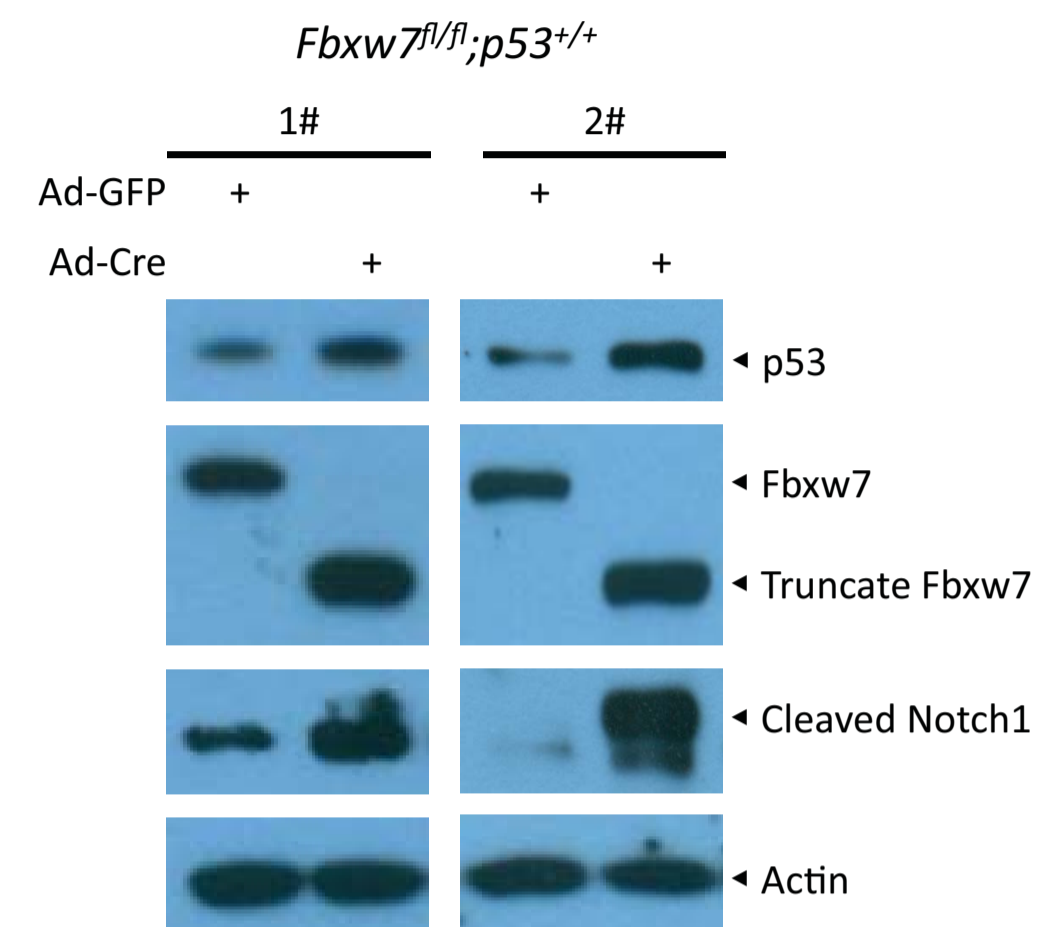

### Figure 3B

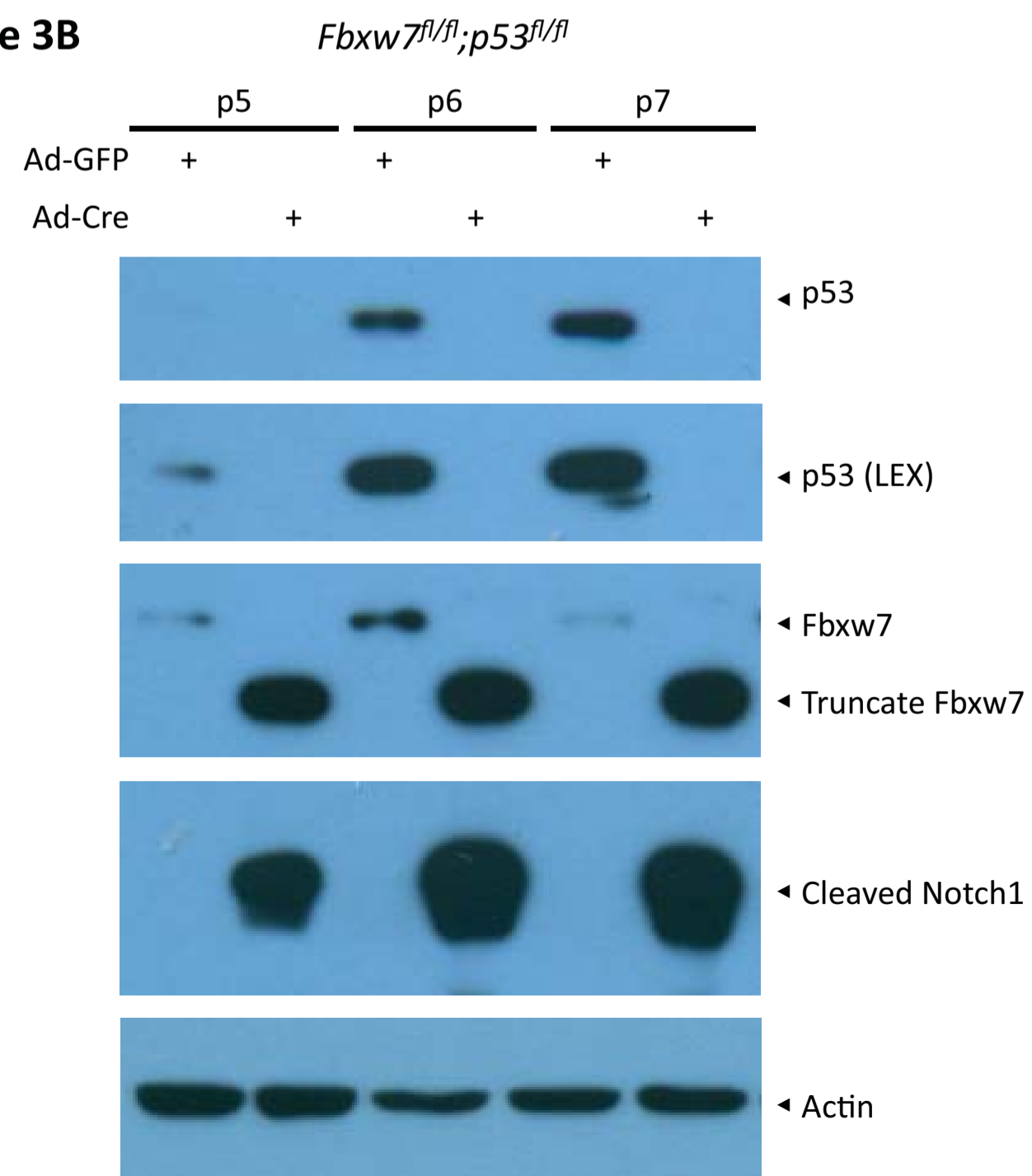

### Figure 4D

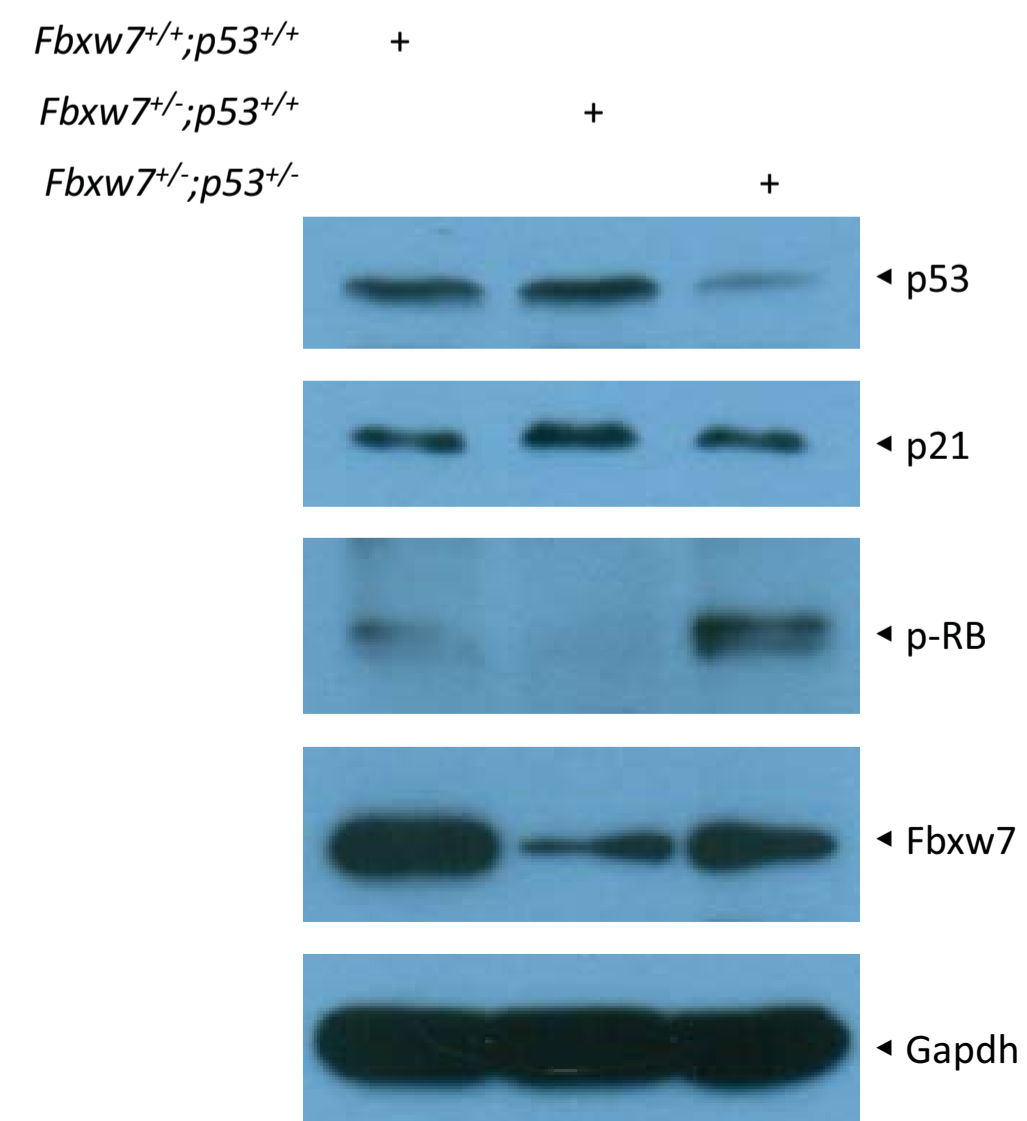

Figure S1A

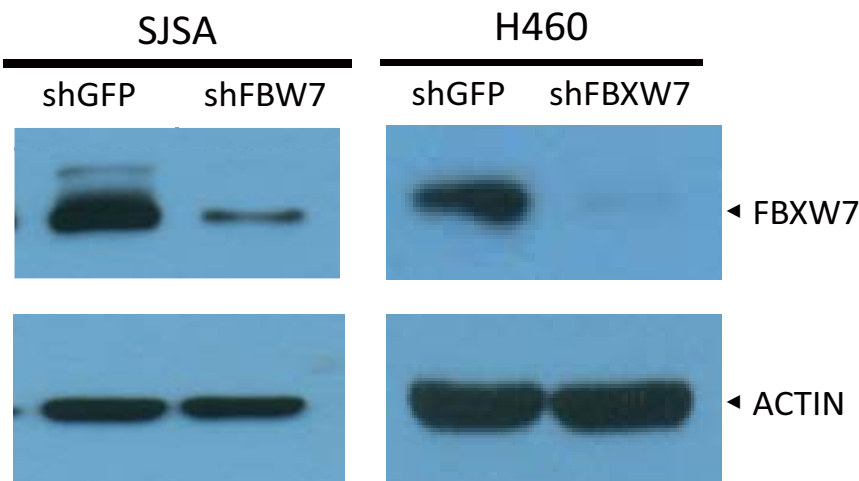

Figure S2A

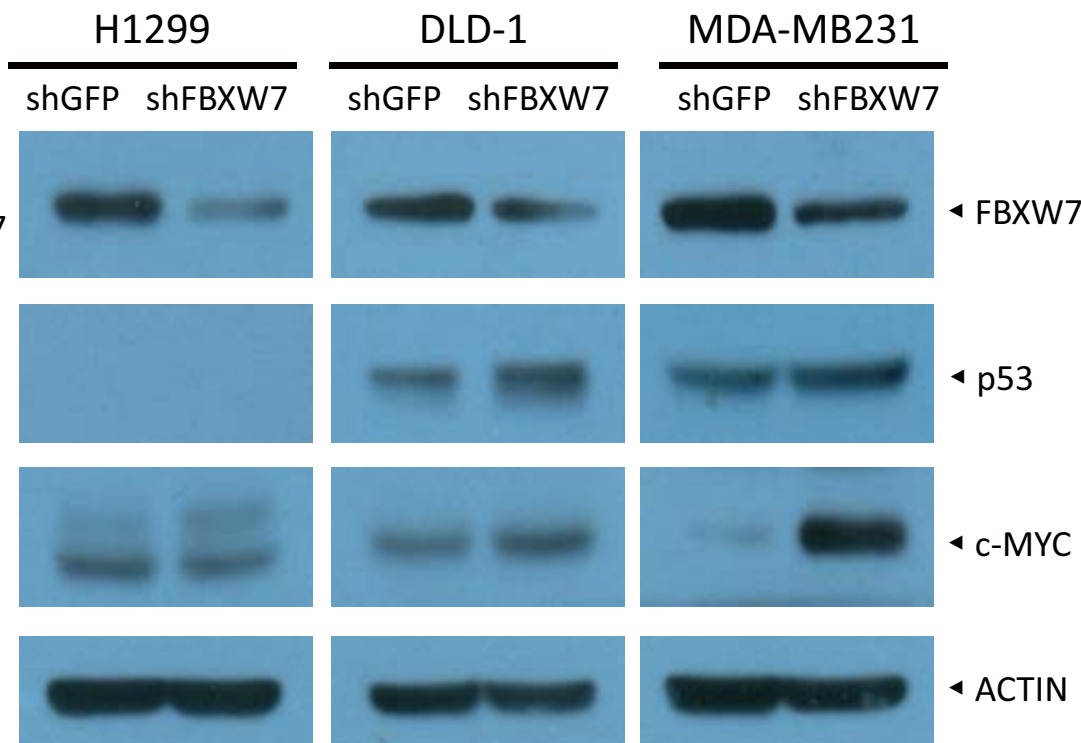

Figure S3A

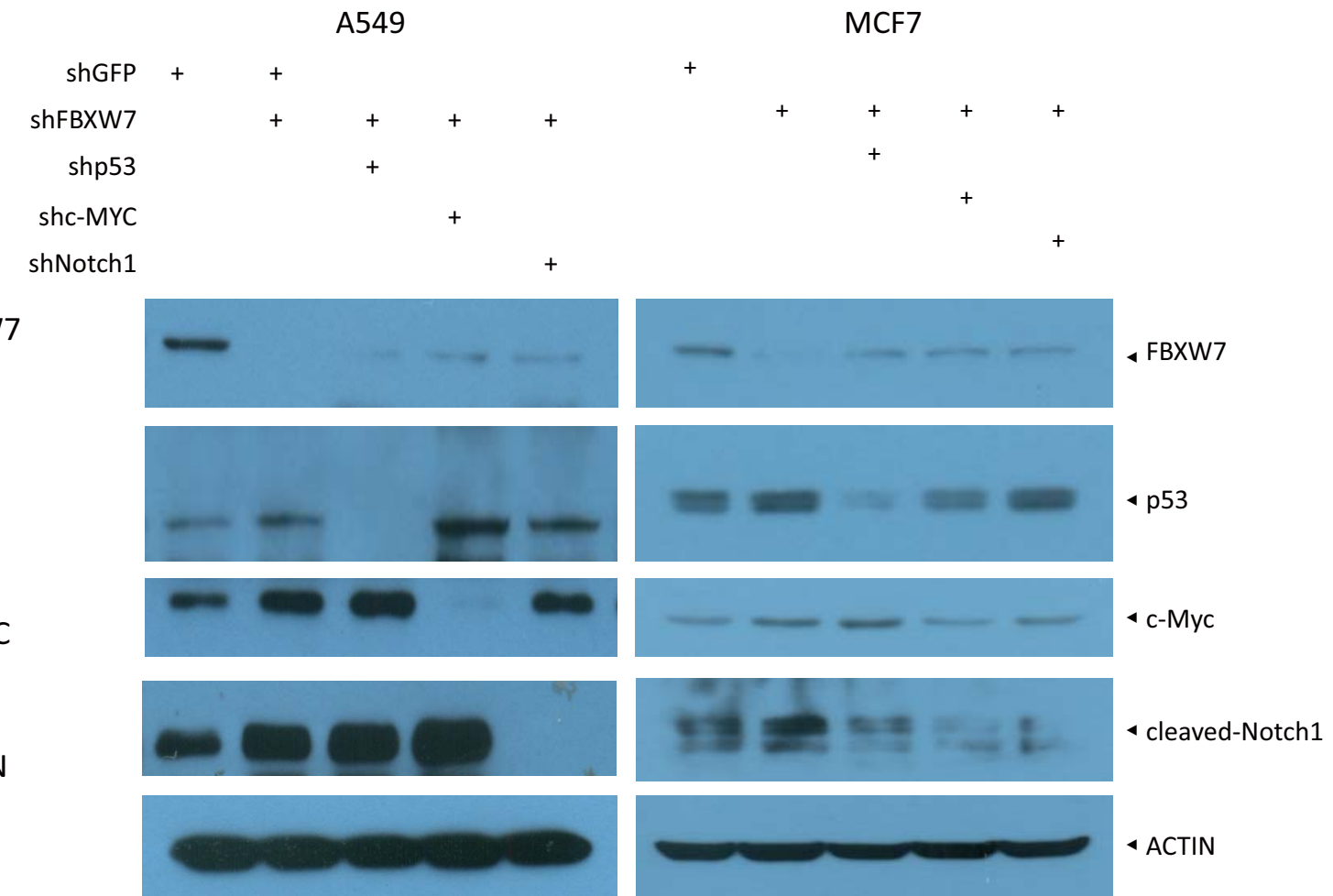

Supplement: Supplementary file 5 — Original IBs [file 41419_2022_5229_MOESM5_ESM.pdf]
