# Supplementary material for: FBXW7 inactivation induces cellular senescence via accumulation of p53
Source: Cell Death Dis. 2022 Sep 14;13(9):788. doi: 10.1038/s41419-022-05229-2 (PMC9475035; doi:10.1038/s41419-022-05229-2)
Supplement: Supplementary file 4 — Supplementary Figure legends [file 41419_2022_5229_MOESM4_ESM.docx]

**Supplemental information**

**Supplemental figure legends**

**Figure S1. FBXW7 silencing induces senescence in cancer cells harboring wild-type p53.**

SJSA and H460 cells were infected with a lentivirus expressing shFBXW7 or shGFP, selected by puromycin for seven days, and subjected to immunoblotting (IB) with the indicated antibodies (A) or senescence-associated β-galactosidase (SA-β-Gal) staining, followed by microscopy (B, left). The percentage of SA-β-Gal-positive cells in the total number of cells was determined (B, right). (C) A549, HCT116, and MCF7 cells were infected with a lentivirus expressing shFBXW7 or shGFP, selected by puromycin for seven days, and subjected to Sudan-Black-B (SBB) staining, followed by microscopy. Scale bars represent 50 μm. Data are presented as the mean ± standard deviation (SD). ****p* < 0.001.

**Figure S2. FBXW7 silencing has little, if any, effect on the induction of senescence in cancer cells harboring null/mutant p53.**

H1299, DLD-1, and MDA-MB231 cells were infected with a lentivirus expressing shFBXW7 or shGFP, selected by puromycin for seven days, and subjected to IB with the indicated antibodies (A) or SA-β-Gal staining, followed by microscopy (B, left). The percentage of SA-β-Gal-positive cells in the total number of cells was determined (B, right). Scale bars represent 50 μm. Data are presented as the mean ± SD. ns: not significant.

**Figure S3. Simultaneous p53 silencing abrogates the senescence induced by FBXW7 knockdown**

A549 and MCF7 cells were infected with a lentivirus expressing indicated shRNAs, selected by puromycin for seven days, and subjected to IB with indicated antibodies (A), or SA-β-Gal staining followed by microscope (B). The percentage of SA-β-Gal positive cells in the total number of cells was determined (B, right). Scale bars represent 50 μm. Data are presented as mean ± SD. *** *p* < 0.001.
